# Serial Examination of an Inducible and Reversible Dilated Cardiomyopathy in Individual Adult *Drosophila*

**DOI:** 10.1371/journal.pone.0007132

**Published:** 2009-09-22

**Authors:** Il-Man Kim, Matthew J. Wolf

**Affiliations:** Department of Medicine, Duke University, Durham, North Carolina, United States of America; University of Texas MD Anderson Cancer Center, United States of America

## Abstract

Recent work has demonstrated that *Drosophila* can be used as a model of dilated cardiomyopathy, defined as an enlarged cardiac chamber at end-diastole when the heart is fully relaxed and having an impaired systolic function when the heart is fully contracted. Gene mutations that cause cardiac dysfunction in adult *Drosophila* can result from abnormalities in cardiac development or alterations in post-developmental heart function. To clarify the contribution of transgene expression to post-developmental cardiac abnormalities, we applied strategies to examine the temporal and spacial effects of transgene expression on cardiac function. We engineered transgenic *Drosophila* based on the well-characterized temperature-sensitive Gal80 protein in the context of the bipartite Gal4/UAS transgenic expression system in *Drosophila* employing the cardiac specific driver, tinCΔ4-Gal4. Then, we developed a strategy using optical coherence tomography to serially measure cardiac function in the individual flies over time course of several days. As a proof of concept we examined the effects of the expression of a human mutant delta-sarcoglycan associated with familial heart failure and observed a reversible, post-developmental dilated cardiomyopathy in *Drosophila*. Our results show that the unique imaging strategy based on the non-destructive, non-invasive properties of optical coherence tomography can be applied to serially examine cardiac function in individual adult flies. Furthermore, the induction and reversal of cardiac transgene expression can be investigated in adult flies thereby providing insight into the post-developmental effects of transgene expression.

## Introduction

The identification of genes that cause or modify cardiac function is important in understanding the complex biology that is responsible for dilated cardiomyopathy and heart failure in humans. Recent work has demonstrated that *Drosophila* can be used as a model of cardiovascular disease.[1], [2], [3], [4] We have developed a strategy using optical coherence tomography (OCT) imaging in awake, adult *Drosophila* to identify genes that cause dilated cardiomyopathy, defined as an enlarged cardiac chamber at end-diastole when the heart is fully relaxed and impaired systolic function when the heart is fully contracted.[4] Optical coherence tomography (OCT) is based on reflectivity of near-infrared light and provides functional image information of the adult fly heart in a manner similar to echocardiography in humans.[4], [5] Although OCT has limited spacial resolution and the evaluation of the adult *Drosophila* cardiac chambers is limited to the conical chambers located in the A1–A2 segments, OCT imaging is well-suited as a strategy to systematically evaluate the impact that gene mutations have on the development of dilated cardiomyopathy in the adult fly.

Since the cardiac abnormalities associated with mutations or transgene expression can result from alterations in embryonic dorsal vessel development or manifest later in the mature adult heart, we sought to develop a strategy to examine post-developmental temporal effects of cardiac-specific transgene expression. To test this strategy, we examined a human mutation in delta-sarcoglycan (SGCD) as a proof-of-concept.

We previously demonstrated that the expression of human mutation in SGCD with a serine to alanine mutation at amino acid position 151 (S151A) previously associated with human familial dilated cardiomyopathy results in the dilated cardiomyopathy in awake, adult flies whereas the human wild-type SGCD does not affect *Drosophila* cardiac function [4], [6]. The expression of the SGCD transgenes were under the control of tinCΔ4-Gal4 that is driven in *Drosophila* cardioblasts from early dorsal vessel development and through adulthood [7]. This observation raised the question regarding the temporal relationship between mutant transgene expression and the cardiac dysfunction that was observed in the adult fly. We used a set of transgenic *Drosophila* based on the well-characterized temperature-sensitive Gal80 protein (Gal80^ts^) in the context of the bipartite Gal4/UAS transgenic expression system in *Drosophila* employing the cardiac specific driver, tinCΔ4-Gal4. [7], [8], [9], [10], [11], [12] Then, we modified our use of OCT in a unique manner to perform a serial evaluation of the cardiac function in individual awake, adult *Drosophila* of the course of several days.

## Results

To test whether post-developmental, cardiac expression of transgenes can cause dilated cardiomyopathy, we engineered a driver line for the inducible, cardiac specific expression of transgenes based on the p{tub-Gal80^ts^} system described by McGuire *et. al.* that examined *Drosophila* transgene expression in the nervous system.[9], [10] We then examined the post-developmental effects of a mutant S151A-human-SGCD that has been previously shown to cause dilated cardiomyopathy in *Drosophila*. We examined p{tub-Gal80^ts^}; p{tinCΔ4-Gal4} flies that harbored either p{UAS-wt-human-SGCD} or p{UAS-S151A-human-SGCD}. Since the context of the genetic background is known to influence the phenotypic expression of traits, we used double homozygous p{tub-Gal80^ts^}; p{tinCΔ4-Gal4} as a stock and then crossed in specific UAS-transgenes that were engineered in a *w^1118^* background. We examined the F1 flies from these crosses so that the genetic backgrounds were similar between groups for all investigations. As a control, we examined the cardiac function in the double homozygous p{tub-Gal80^ts^}; p{tinCΔ4-Gal4} driver line and determined that the cardiac parameters measured by OCT were similar to *w^1118^* and *Oregon-R* (Table 1). Additionally, we previously demonstrated that the effect of UAS-human SGCD was independent of the transgene insertion location within the genome since multiple insertion lines had similar effects on cardiac function[4]. Furthermore, since individual flies were examined in a serial manner, each baseline cardiac assessment served as a control for subsequent measurements.

**Table 1:** Cardiac parameters in *w^1118^, Oregon-R*, and the cardiac-specific, inducible driver line.

	N	EDD (microns)	ESD (microns)	FS (%)
*W^1118^*	11	70±5	3±1	96±2
*Oregon-R*	11	75±3	2±1	97±2
Gal80^ts^;tinCΔ4-Gal4 driver line	12	76±4	3±1	96±2
Gal80^ts^; tinCΔ4-Gal4 harboring WT-SGCD	16	64±2	1±1	99±1
Gal80^ts^; tinCΔ4-Gal4 harboring S151A-SGCD	16	63±4	2±1	97±1

OCT measurements of end diastolic dimension (EDD) in microns, end systolic dimension (ESD) in microns, and fractional shortening (FS) in percentage are expressed as the mean +/− SE. The values for *w^1118^* (n = 11), *Oregon-R* (n = 12), the homozygous Gal80^ts^; tincΔ4-Gal4 driver line (n = 11) were obtained from flies maintained at 26°C. The values for the Gal80^ts^; tincΔ4-Gal4 driver line harboring either wt-human-SGCD (n = 16) or S151A-human-SGCD (n = 16) were obtained from flies maintained at 18°C.

In our experiments, the Gal80^ts^ protein is functional at 18°C and binds to Gal4 thereby preventing the interaction between Gal4 and the UAS promoter so that the expression of the human-SGCD transgene is repressed. However, at 26°C, Gal80^ts^ dissociates from Gal4 thereby allowing Gal4 to bind to the UAS promoter thereby resulting in human-SGCD transgene expression. Flies were maintained at 18°C until 7 days after eclosion and then baseline cardiac function was examined by OCT. OCT provides detailed measurement of heart rate, end-diastolic dimension (EDD), end-systolic dimension (ESD), and fractional shortening (FS), a surrogate measurement of contractility. Since OCT is non-destructive and non-invasive, we recovered each fly after image acquisition and repeated serial assessments of cardiac function. The flies were maintained at 26°C and cardiac function in individual flies was serially re-examined at 24, 48, and 96 hours after baseline cardiac assessment. Then, the flies were returned to 18°C and cardiac function was serially examined at 120 and 168 hours after baseline cardiac assessment. During the same time course, parallel groups of flies were used to examine human-SGCD transgene expression using QRT-PCR.

**Figure 1:**
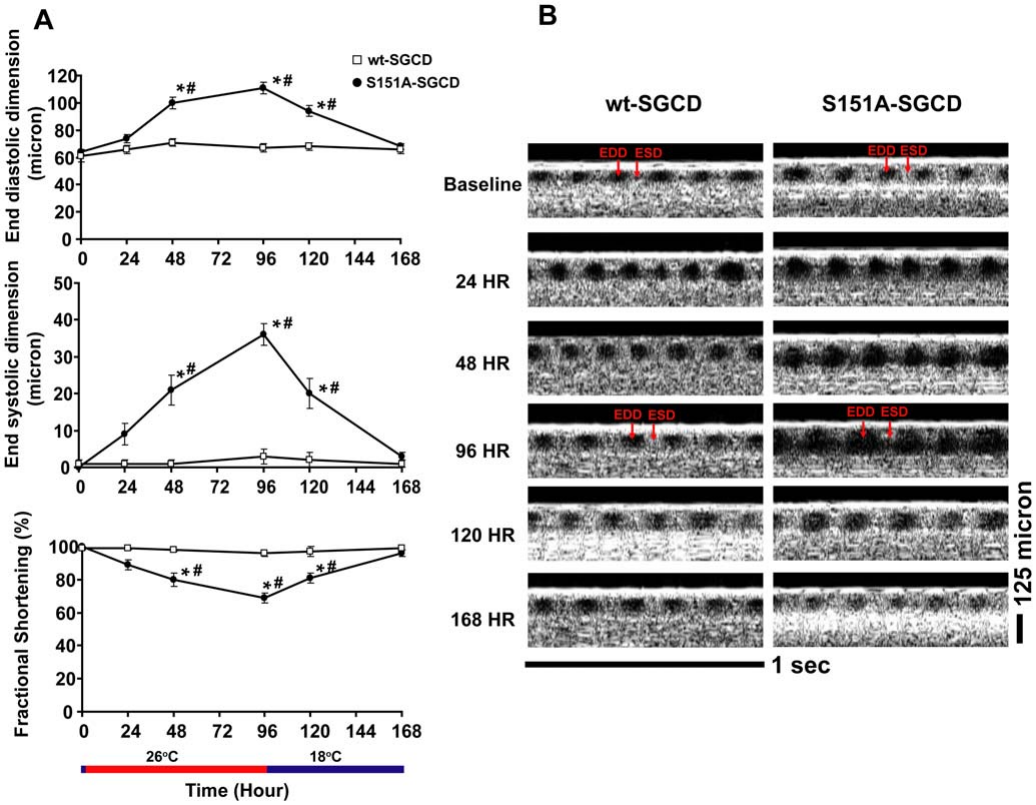
Expression of human mutant S151A-SGCD causes an inducible and reversible dilated cardiomyopathy in adult *Drosophila*. (A) Temperature shift from 18°C to 26°C causes the induction of S151A-SGCD expression and subsequent deterioration in cardiac function. At 96 hours post induction, flies expressing S151A-SGCD demonstrate an enlargement in EDD and ESD with a resultant impairment in FS. At 96 hours, a temperature shift back to 18°C results in a repression of S151A-SGCD expression and subsequent improvement in cardiac function with return of EDD, ESD and FS to near baseline. A similar level of wt-SGCD expression after temperature shift from 18°C to 26°C does not result in deterioration in cardiac function. Each graph represents the summary data for serial OCT measurements of EDD, ESD and FS and are expressed as the mean +/− SE (n = 16 for wt-SGCD and n = 16 for S151A-SGCD). * P<0.05 for time point measurements compared to baseline and *#* P<0.05 for measurements between wt-SGCD and S151A-SGCD at each time point. (B) Representative serial OCT m-modes in individual flies expressing S151A-SGCD or wt-SGCD at the indicated times and temperatures. The arrows indicate EDD when the fly heart is fully relaxed and ESD when the fly heart if fully contracted. A 125 micron standard and 1 second scale bar is shown.

The expression of wt-human-SGCD had no significant effect on cardiac function since the cardiac parameters remained stable throughout the experiments in the F1 offspring from a cross between p{tub-Gal80^ts^}; p{tinCΔ4-Gal4} flies and flies that harbored p{UAS-wt-human-SGCD} (Figure 1). Fractional shortening was 99+/−1% in flies harboring p{UAS-wt-human-SGCD} at 18°C and 96+/−2% at 26°C for 96 hours. Additionally, the cardiac function at 18°C in the F1 offspring from a cross between p{tub-Gal80^ts^}; p{tinCΔ4-Gal4} flies and flies that harbored either p{UAS-wt-human-SGCD} or p{UAS-S151A-human-SGCD} was similar to the common laboratory stock, *w^1118^* and Oregon-R (Table 1).

**Figure 2:**
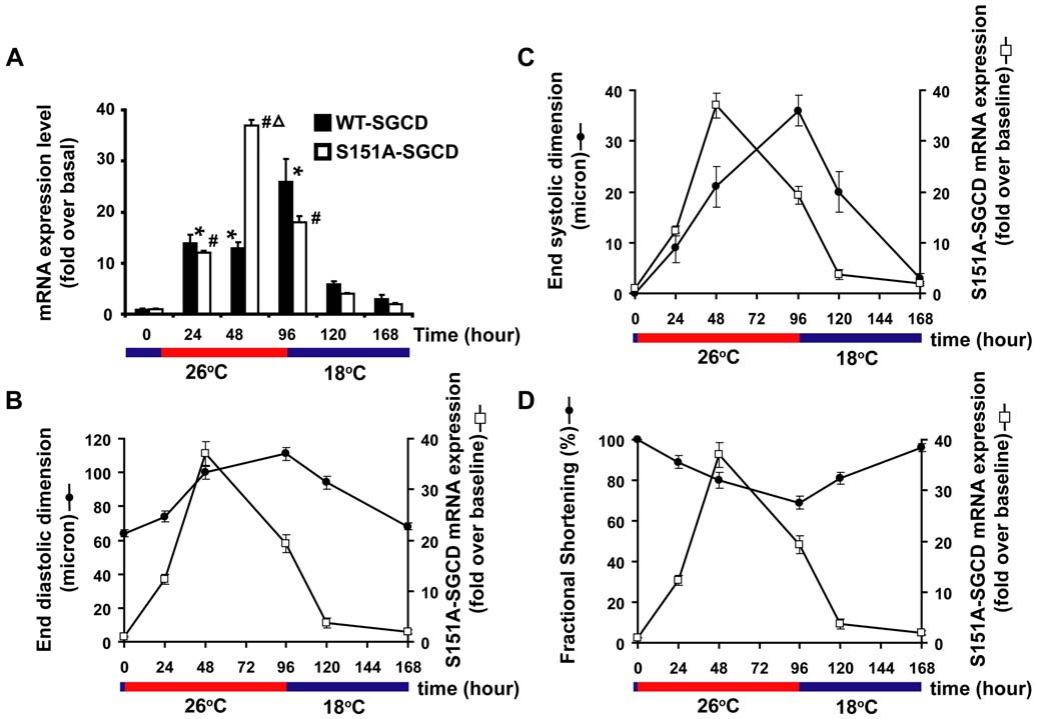
Expression of S151A-SGCD precedes cardiac function changes in adult *Drosophila* expressing S151A-SGCD. (A).Temperature shift from 18°C to 26°C results in deterioration in cardiac function and induction of S151A-SGCD expression. After a temperature shift to 26°C, the induction of transgene expression precedes the development of dilated cardiomyopathy by 48 hours as demonstrated by QRT-PCR measurements of S151A-SGCD mRNA levels compared to cardiac function by serial OCT. A second temperature shift back to 18°C represses S151A-SGCD mRNA expression and results in restoration of cardiac function by 48 hours. We performed three independent experiments using different batches of flies each time. The summary data for QRT-PCR at the indicated times and temperatures are expressed as the mean +/− SE of three independent experiments, each performed in triplicate. * p<0.05 for S151A-SGCD expression at indicted time vs. baseline; # p<0.05 for wt-SGCD expression at the indicated time points vs. baseline; Δ p<0.05 for wt-SGCD vs. S151A-SGCD at the 48 hour time point. (B–D) Summary data for the time course of S151A-SGCD expression (closed circles) vs. cardiac measurements (closed squares) based on serial measurements of EDD, ESD, and FS as described in Figure 1.

Interestingly, the F1 offspring from a cross between p{tub-Gal80^ts^}; p{tinCΔ4-Gal4} flies and flies that harbored p{UAS-S151A-human-SGCD} demonstrated a progressive deterioration in cardiac function manifest as an increase in ESD and EDD with a resultant decrease FS. By 96 hours, FS decreased from 97+/−1% to 69+/−3%; p<0.05 (Figure 1). The changes in cardiac function paralleled increases in human-SGCD transgene expression but were delayed by about 24 hours after the induction of transgene expression as determined by QRT-PCR of human SGCD (Figure 2). Moreover, a temperature shift from 26°C to 18°C resulted in a significant reduction in SGCD transgene expression to baseline levels and concomitant improvement in cardiac function with a restoration of ESD, EDD, and FS to baseline values in the S151A- SGCD group. Again, the improvement in cardiac function paralleled the reduction in transgene expression with functional changes lagging transgene expression changes by about 24 hours as determined by QRT-PCR (Figure 2).

## Discussion

Our results demonstrate that serial cardiac measurements can be easily obtained in adult *Drosophila* and that we can examine the post-developmental effects of transgene expression and repression on cardiac function. To date, the assessment of cardiac function in adult flies has mostly relied on an examination of partially dissected specimens that are perfused in a temperature-controlled artificial hemolymph solution.[1], [13], [14] While this approach provides valuable information about cardiac function, the analysis of a partially dissected preparation is confined to several hours until the specimen deteriorates. Thus, an approach that is based on the examination of dissected heart preparations precludes a serial examination of the same fly over an extended period of time.

For the first time, we demonstrate that our non-invasive imaging strategy has the advantage of examining cardiac function over the course of several days and possibly weeks. This provides the possibility to serially examine age-related changes in cardiac function in individual flies where each fly can be evaluated by repeated measurements, a technique that is applicable to mouse models of cardiac disease but thus far not available to fly models. Additionally, our strategy is applicable to a variety of transgenes including flies that harbor si-RNA against a single protein coding gene. Furthermore, the cardiac effects of toxins, drugs, and other environmental exposures can be examined using this strategy.

We chose to examine a mutant human-SGCD in our study as a proof-of-concept to address the concept that post-developmental cardiac abnormalities can result from the temporal regulation of transgene expression. Interestingly, we not only observed that the cardiac function in adult flies declined in response to the expression of the mutant S151A-human-SGCD but that subsequent transgene repression resulted in an improvement in cardiac function. Delta-sarcoglycan is a component of the dystrophin glycoprotein complex located on the plasma membrane of skeletal and cardiac myocytes.[15] The dystrophin glycoprotein complex is required for normal muscle cell functions and is involved in the transmission of signals from the extracellular matrix to the contractile machinery within the cell. The S151A-human-SGCD may act as a dominant negative isoform.[6] Accordingly, the expression of the S151A-SGCD may disrupt or, at least, alter the proper functioning of dystrophin glycoprotein complex thereby inhibiting extracellular signals that are necessary to maintain normal myocyte function. Conversely, the repression of S151A-SGCD removes the dominant-negative effect and allows restoration of the endogenous dystrophin glycoprotein complex. Interestingly, we observed that the changes in cardiac function occurred about 24 hours after transgene expression was induced or repressed, a time course that appears consistent with transgene protein production and incorporation into endogenous dystrophin glycoprotein complex. While the exact mechanisms that are responsible for the deterioration and subsequent improvement in cardiac function are not known,mutations that disrupt the sarcoglycan complex have been associated with dilated cardiomyopathies in flies and mammals[4], [16], [17].

Collectively, our results suggest that the combination of the established power of the Gal80^ts^ system in conjunction with the unique properties of OCT can provide a reliable strategy to examine the temporal effects of transgene expression on cardiac function in the adult fly heart. We believe that the development of this methodology should provide insight into signaling pathways that are responsible for post-developmental cardiac dysfunction and may lead to insights into reparative mechanisms for human heart failure.

## Materials and Methods

### Fly stocks

w[*]; P{tubP-GAL80[ts]}10; TM2/TM6B, Tb[1] was obtained from the Bloomington *Drosophila* Stock Center. Transgenic p{UAS-WT-human-SGCD} and p{UAS-S151A-human-SGCD} flies were engineered in w1118 as previously described[4]. We obtained two transgenic lines for p{UAS-WT-human-SGCD} and three transgenic lines for p{UAS-S151A-human-SGCD}. In our experiments, we used stocks for each transgene that demonstrated similar levels of transgene expression at baseline. The p{tincΔ4-gal4} fly line was generated in a yw background and kindly proved by Manfred Frasch[12]. For all experiments, we examined the F1 offspring of crosses between the double homozygous P{tubP-GAL80[ts]}10; p{tincΔ4-gal4} and either p{UAS-WT-human-SGCD} or p{UAS-S151A-human-SGCD}. All fly stocks were maintained on standard yeast protein, carbohydrate, agar media at the indicated temperatures (18°C or 26°C) according to standard procedures.

### Cardiac measurements in adult *Drosophila*

Cardiac function in adult *Drosophila* was measured using a custom built OCT microscopy system (Bioptigen, Inc) as previously described.[4] Briefly, adult *Drosophila* at 7 days post eclosion were briefly subjected to CO2, placed on a soft gel support, and allowed to fully awaken based on body movement. Multiple OCT m-modes of heart function from awake *Drosophila* were recorded along with a 125 micron standard. All images were acquired as. OCT files using Bioptogen software and then processed using ImageJ software. End-diastolic (EDD), end-systolic (ESD), and heart rate were determined from 3 consecutive heart beats. Fractional shortening (FS) was calculated as [EDD-ESD]/EDD x 100. All OCT imaging was conducted at room temperature and flies were immediately returned to the designated experimental temperatures after imaging.

For serial OCT studies, adult flies were bred at 18°C until 7 days post eclosion and baseline cardiac measurements were obtained using OCT. Each fly was gently removed from the soft gel support and placed in individual vials and maintained at 26°C. Cardiac function in each individual fly was repetitively measured at 24, 48, and 96 hours. Then, flies were returned to 18°C and cardiac function was imaged at 120 and 168 hours after baseline cardiac assessment.

### QRT-PCR

Total RNA from thirty flies at each of the indicated times and temperatures was prepared using RNA-Bee (Tel-Test “B,” Inc., Friendswood, TX) and treated with RNase-free DNaseI to remove genomic DNA. cDNA was synthesized using invitrogen (Carlsbad, CA) SuperScript II reverse transcriptase. Applied Biosystems Taqman Gene expression assays were used to perform quantitative (real time) RT-PCR (human delta-sarcoglycan, Hs01087180_m1 and fly ribosomal protein L32, Dm02151827_g1 for endogenous control). The following reaction components were used for each probe: 2 uL cDNA, 12.5 ul 2X TaqMan Universal PCRMaster Mix (Applied Biosystems, Foster City, CA), 1.25 ul of either Hs01087180_m1 or Dm02151827_g1 probe, and 9.25 ul water in a 25 µL total volume. Reactions were amplified and analyzed in triplicate using an ABI PRISM® 7000 Sequence Detection System. PCR reaction conditions were as follows: Step 1: 50°C for 2 minutes, Step 2: 95°C for 10 minutes, Step 3: 40 cycles of 95°C for 15 seconds followed by 60°C for 1 minute. Expression relative to ribosomal protein L32 was calculated using 2^−ΔΔCt^ and levels were normalized to baseline. We performed three independent experiments in triplicate using different batches of flies each time.

### Statistical Analysis

Statistical analysis was performed using GraphPad. Analyses of variance (ANOVA) were performed using Bonferroni corrections for multiple comparisons.
